# Correction: Food and family care during the COVID-19 pandemic: A study of women’s domestic workload during the first wave in Chile

**DOI:** 10.1371/journal.pone.0312393

**Published:** 2024-10-15

**Authors:** Nathalie Llanos, Lorena Iglesias, Patricia Gálvez Espinoza, Carla Cuevas, Dérgica Sanhueza

There are errors in the funding statement. The correct funding statement is as follows: This work was supported by the Ines Género Project at the University of Chile (INGE210028 to PGE, NL, and LIV) and the National Fund for Scientific and Technological Development (Fondecyt) program at the National Agency for Research and Development (ANID) (Grant No.: 11180370 awarded to PGE). The funders had no role in the study design, data collection, analysis, publication decision, or manuscript preparation. No additional external funding was received for this study.
