# Supplementary material for: Food and family care during the COVID-19 pandemic: A study of women’s domestic workload during the first wave in Chile
Source: PLoS One. 2024 May 24;19(5):e0301038. doi: 10.1371/journal.pone.0301038 (PMC11125543; doi:10.1371/journal.pone.0301038)
Supplement: S1 Table — (DOCX) [file pone.0301038.s001.docx]

S1 Table. Items for the three scales and their descriptive statistic

|  | **Score Mean** | **SD** |
| --- | --- | --- |
| **Spending on food and food purchases dimension** | | |
| I feel like I spend more money on food. | 2,29 | 1,369 |
| I feel that it is more challenging to organize the budget for food. | 2,97 | 1,351 |
| I feel like I've had to buy more food. | 2,19 | 1,336 |
| I feel like it has become more difficult for me to go out and buy food. | 2,07 | 1,344 |
| I feel that I have used apps, to a greater extent, to buy food and bring it to my home | 2,69 | 1,578 |
| I feel like I spend more time shopping for groceries. | 2,89 | 1,430 |
| I feel like I've bought more food than I really need. | 2,81 | 1,389 |
| I think I should look for more strategies to make the budget work. | 3,10 | 1,357 |
| **Food preparation dimension** | | |
| I feel like I cook more than before | 1,95 | 1,291 |
| I feel like I spend more time in the kitchen than before. | 1,94 | 1,271 |
| I feel like I can't stop washing dishes | 2,22 | 1,353 |
| I feel like now I have to cook different types of meals every day. | 2,26 | 1,370 |
| I feel like I spend all day preparing food. | 2,91 | 1,293 |
| I feel like I spend more time thinking about what meals to prepare. | 2,35 | 1,309 |
| I feel like I have had to put aside my personal activities because I have to cook. | 2,88 | 1,346 |
| I feel like I have to put aside my work to prepare food. | 3,06 | 1,327 |
| I feel like I have been able to balance cooking well with other personal activities. | 3,33 | 1,196 |
| I feel like I've made more home-cooked meals these days. | 4,07 | 1,192 |
| I feel like I have bought more meals prepared outside the home (for example, from some restaurant or another) | 4,01 | 1,093 |
| I feel like I eat healthier now. | 3,30 | 1,297 |
| I feel overwhelmed by everything that needs to be done in the kitchen. | 3,08 | 1,345 |
| **Family care dimension** | | |
| I feel like I spend more time worrying about others in my family. | 2,39 | 1,259 |
| I feel like I do more activities with my family. | 3,49 | 1,248 |
| I feel that I should take more care of my family's nutrition. | 2,28 | 1,243 |
| I feel like I should be more aware of my family's eating routines. | 2,35 | 1,252 |
| I feel like my family's eating routines have gotten worse. | 3,74 | 1,128 |
| I feel like I have to put my job aside to feed my family. | 3,42 | 1,293 |
| I feel like I care more about cooking healthy for my family. | 2,49 | 1,299 |
| I feel like I am more aware of the nutrition of others in my family. | 2,73 | 1,334 |
| I feel that the hours of the day are not enough to fulfill household chores and paid work | 2,56 | 1,472 |
| I feel that home activities are more important than paid work activities. | 3,09 | 1,250 |
| I feel that I have balanced the activities at home with those needed in other areas of my life. | 3,13 | 1,262 |
| I feel like I'm more stressed now with all the housework. | 2,57 | 1,372 |
| I feel like I should be much more aware of others in my family. | 2,54 | 1,289 |
| I feel overwhelmed with all the work that comes with the house | 2,81 | 1,367 |
