# Supplementary material for: Food and family care during the COVID-19 pandemic: A study of women’s domestic workload during the first wave in Chile
Source: PLoS One. 2024 May 24;19(5):e0301038. doi: 10.1371/journal.pone.0301038 (PMC11125543; doi:10.1371/journal.pone.0301038)
Supplement: Attachment — Submitted filename: Responses to reviewers.pdf [file pone.0301038.s003.pdf]

Responses to reviewers

We appreciate the reviewers for your precious time in reviewing our paper and providing valuable comments. We have carefully considered the comments and tried our best to address every one of them.

Below, we provide the point-by-point responses. All modifications in the manuscript have been highlighted in red.

Reviewer #2

Unfortunately there are no improvements marked in different color, or with track changes function. Still i was not able to detect significant improvements to the paper.

Response: We are sorry you did not receive the correct version of our manuscript. According to journal requirements, we uploaded two versions of our document: one with changes made to it in red and another one with no marks. We do not know what could happen. We have uploaded two versions again, including one with changes in red.

While the topic was relevant at the time of the research thousands of papers with more robust analysis have come out since 2020.
The research is not sufficiently original in its current form to merit publication in top tier journals.

Response: We thank the reviewer for allowing us to reflect on this issue. We agree that there is vast evidence about different topics in the COVID-19 context. However, our study has several reasons for being published in a journal such as PLOS ONE.

1. There needs to be more evidence of the domestic burden on women in Latin America, especially regarding the domestic food environment in times of crisis.
2. The sample size is superior to other studies in the field during the pandemic.
3. This study incorporates quantitative and qualitative. The methodology used in this study may differ and offer a different or complementary vision to the most recent research.
4. As the COVID-19 pandemic was a new global situation, it was a natural experiment for several social aspects in people's lives, including food and the food environment. This research could provide evidence about what happens in health and social crises in women's domestic work, which could be considered in future similar scenarios.
5. Despite the time that has passed, the impact of the pandemic on housework remains a crucial topic of general interest, which makes this study maintain its relevance. Before the pandemic, there were gender role inequalities, and the COVID-19 crisis aggravated them. Today, no concrete measures have been taken to reverse this situation, remaining as a relevant issue.

6. This study could work as a baseline for future research that could evaluate what happens in a post-pandemic period with the variables exposed here.

This study can contribute to the current conversation, complement new research with its approach, and give information to face future similar crises. We added some of these reflections to our manuscript.

Reviewer #3

The subject of the article, the increase in gender inequality as a result of the first COVID wave in Chile, is a highly interesting one. The online survey offered to Chilean women could have yielded new and robust data if it had included a control arm, i.e. a similar survey offered to Chilean men.

Response: We appreciate your perspective on the importance of including a balanced perspective when addressing the rise in gender inequality during the Chilean first wave of the COVID-19 pandemic. Having comparative data between men and women would have considerably enriched our analysis. However, we decided to work only with women because, in Chile, it is known that women have a more significant burden in domestic work, especially in tasks related to feeding inside the home.

In future research, including a representative sample of men to understand their perspective and contribution to household responsibilities during a health crisis would be relevant to obtain a complete and balanced picture of how the crisis affected both genders. Also, it would be interesting to investigate different family structures comparatively to know if some structures have more burden on domestic work.

Your suggestion is valuable, and we consider that an inclusive approach in future research in this area would be essential to address the changes in gender dynamics in times of crisis more holistically, like the one we have gone through. We added some of these reflections to our manuscript. Please refer to lines 457 to 461.

In its methodology, this survey can only produce descriptive results, the most interesting of which seems to me to be the high percentage of women who perceived themselves to be in poorer health during the months of May and June 2020, compared with an earlier period.

Response: Thanks the reviewer for letting us reflect on this issue. We acknowledge that our study had several limitations. However, we think that most of our results are interesting to understand the phenomenon in a deeper way.

We agree that a relevant one is women perceiving themselves in poorer health status during the studied period. We have improved the discussion about this point, adding possible reasons for this perception according to other studies, among which changes in diet, physical activity and access to medical consultations are mentioned. Please refer to lines 416 to 421.

Logistic regression does not seem to offer any innovative results here. It concludes that younger, better-educated women suffer more from the increase in domestic tasks, but these are women with younger (less autonomous) children on the one hand (which has not been discussed), and who have jobs that they still carry out at a distance + domesticity outside the crisis (which has been discussed).

Response: Thanks to the reviewer for sharing his/her detailed analysis of our research methodology. We recognize the importance of critically evaluating the methods used and the findings presented.

Few studies have worked on changes in domestic work concerning the domestic environment, especially in Latin America. Therefore, the results could be interesting to consider in future health or social crises with similar characteristics.

The reviewer's observations about the influence of children's age and their level of autonomy, as well as the impact of the combination of remote work and domestic responsibilities during the crisis, are very valid points that could play a crucial role in the distribution of the unequal burden of household responsibilities among women of different demographic groups and employment situations. In future studies, exploring these elements and their correlation with increased housework would be essential for a more complete and precise understanding of the dynamics in the food environment. We have added these reflections to the discussion. Please refer to lines 386 to 389.

I agree with the previous reviewer that the main interest of this study lies in its qualitative component, which could be submitted alone (with contextualization by describing the survey population) in a social science journal.

Response: Thanks to the reviewer for letting us reflect on this issue. In general, qualitative analysis is a rich approach to understanding a phenomenon. However, as in this present study, the qualitative analysis just included an analysis of the comments made by some participants; they do not have enough strength to explain the women´s perception of domestic work during the pandemic. We think the phenomenon is better understood using both analysis and results.

On a purely formal level, if the editors were to retain this manuscript for publication:

1. it seems to me that the introduction would have to be reorganized: first part on the global situation of increased domestic tasks linked to confinement, then second part on the situation specific to Chile. Here, following comments from the first reviewers, the two have been merged (ex: reference to Bangladesh in the middle of the focus on Chili).

Response: Thanks to the reviewer for this suggestion. We have reviewed the introduction, reorganized it, and relocated the wrong reference location.

2. Ligne 62: "The sanitary crising DUE TO COVID-19" or "during spring 2020"

Response: Thanks to the reviewer for this suggestion. We have changed the line for "The sanitary crisis due to COVID-19." Please refer to line 64.

3. Lines 73 and 77 of the revised manuscript contradict each other: the crisis has made gender inequalities more visible (line 73) but these inequalities are invisibilized (line 77): it seems to me that the crisis has made them more marked but not more visible.

Response: Thanks to the reviewer for noticing this. We have reviewed this phrases.

4. Line 96: this belongs to the discussion or conclusion

Thanks to the reviewer for this suggestion. We have moved this phrase to the discussion. Please refer to lines 346 to 349.

5. Line 199-200:the results on women's domestic workload are very concise, not tabulated and difficult to understand. insofar as they are not compared with men's domestic workload, i will not present them.

Response: Thanks to the reviewer for this suggestion. We have rewritten this paragraph and added more information about this variable. Please refer to lines 196 to 206. Additionally, we have added supplementary material with all the items and their average score.

6. I don't have a line number for the discussion. There is a comma before ref 5".

Response: We have added the line numbers to the manuscript and fixed the comma position.
