# Supplementary material for: Food and family care during the COVID-19 pandemic: A study of women’s domestic workload during the first wave in Chile
Source: PLoS One. 2024 May 24;19(5):e0301038. doi: 10.1371/journal.pone.0301038 (PMC11125543; doi:10.1371/journal.pone.0301038)
Supplement: Attachment — Submitted filename: Responses to reviewers.docx [file pone.0301038.s002.docx]

**Responses to reviewers**

We appreciate the reviewers for your precious time in reviewing our paper and providing valuable comments. We have carefully considered the comments and tried our best to address every one of them.

Below, we provide the point-by-point responses. All modifications in the manuscript have been highlighted in red

**I. Journal Requirements:**

**Comment #1: When submitting your revision, we need you to address these additional requirements.**

Please ensure that your manuscript meets PLOS ONE's style requirements, including those for file naming. The PLOS ONE style templates can be found at

<https://journals.plos.org/plosone/s/file?id=wjVg/PLOSOne_formatting_sample_main_body.pdf> and

<https://journals.plos.org/plosone/s/file?id=ba62/PLOSOne_formatting_sample_title_authors_affiliations.pdf>.

**Response:** Thank you for letting us know about these requirements. We have reviewed the style requirements and fixed the manuscript accordingly.

**Comment #2: Please provide additional details regarding participant consent. In the ethics statement in the Methods and online submission information, please ensure that you have specified (1) whether consent was informed and (2) what type you obtained (for instance, written or verbal, and if verbal, how it was documented and witnessed). If your study included minors, state whether you obtained consent from parents or guardians. If the need for consent was waived by the ethics committee, please include this information.**

**Response:** Thank you for allowing us to clarify this point. All participants were older than 18 years old. We obtained online consent for participating in the study. Before the survey was displayed, a consent form was shown to all participants, explaining the study. At the end of this form was a question about whether they wanted to participate in the study. If they accepted to participate in the study, they marked yes, and then the survey was displayed. A thankful message was displayed if the answer was negative and the survey ended. This information was re-written to the new version of the manuscript.

**Comment #3: If you are reporting a retrospective study of medical records or archived samples, please ensure that you have discussed whether all data were fully anonymized before you accessed them and/or whether the IRB or ethics committee waived the requirement for informed consent. If patients provided informed written consent to have data from their medical records used in research, please include this information.**

**Response:** Thank you for your comment. We did not use medical records or archived samples.

**Comment #4: Thank you for stating in your Funding Statement:**

“This work was partially supported by the National Agency for Research and
Development (ANID) and its National Fund for Scientific and Technological Development (Fondecyt) program, (grant number: 11180370) (PGE), and by the Health Research Grant 2016, from the College of Medicine, Universidad de Chile (PGE and LIV).
The funders had no role in study design, data collection and analysis, decision to publish, or preparation of the manuscript.”

Please provide an amended statement that declares *all* the funding or sources of support (whether external or internal to your organization) received during this study, as detailed online in our guide for authors at <http://journals.plos.org/plosone/s/submit-now>.  Please also include the statement “There was no additional external funding received for this study.” in your updated Funding Statement.

Please include your amended Funding Statement within your cover letter. We will change the online submission form on your behalf.

**Response:** Thank you for letting us amend our funding statement. We have rewritten it and added it to the cover letter.

**Comment #5: We note that the grant information you provided in the ‘Funding Information’ and ‘Financial Disclosure’ sections do not match.**

When you resubmit, please ensure that you provide the correct grant numbers for the awards you received for your study in the ‘Funding Information’ section.

**Response:** Thank you for letting us know about this mistake. We have fixed the funding information.

**II. Reviewers' comments**

**REVIEWER #1:**

**I have some (small) comments that I think would allow to clarify some points:

Comment #1. Regarding previous literature, there are some papers worth mentioning. They are papers for LAC countries analyzing the impact of the pandemic on gender differences in labor outcomes and its connection with care work and gender difference in time use:
- Costoya, V., Echeverría, L., Edo, M., Rocha, A. y Thailinger, A. (2022). “Gender Gaps within Couples: Evidence of Time Re allocations during COVID 19 in Argentina.” Journal of Family and Economic Issues, 43: 213–226.
- Cucagna, E. y Romero, J. (2021). “The gendered impacts of COVID-19 on labor markets in Latin America and the Caribbean.” World Bank Gender Innovation Lab for Latin America and the Caribbean Policy Brief.
- Romero, T.H. y Reys, A. (2020). “Empobrecimiento de los hogares y cambios en el abastecimiento de alimentos por la COVID-19 en Lima, Perú.” Revista de Recursos en Internet sobre Geografía y Ciencias Sociales.
- Viollaz, M., Salazar-Saez, M., Flabbi, L., Bustelo, M. y Bosch, M. (2022). “The COVID-19 Pandemic in Latin American and Caribbean Countries: The Labor Supply Impact by Gender.” IZA Discussion Paper 15091.**

**Response:** Thank you very much for your recommendation. We have reviewed and incorporated these studies into the manuscript. Please, refer to the introduction and discussion section.

**Comment #2 Considering the previous point, the authors may want to stress more the fact that they are analyzing self-perceptions of domestic work, while previous literature considers time use information or whether women/men perform care activities.**

**Response:** Thank you very much for the comment. In the discussion section, we have added a final paragraph where it is expressed as a limitation of the study that the data have been self-reported.

**Comment #3. It would be good to clarify whether the dependent variable in the regression analysis is defined as =1 if the score is "bad" or whether it is =1 if the score is "bad" or "regular".**

**Response**: Thank you very much for allowing clarify this aspect. As the reviewer indicated, the dependent variable was the women's perception of domestic work related to food and family care during the COVID-19 pandemic. None participant perceived their domestic work as "good." Therefore, we defined 1 as the "bad" category in the regression analysis.

**REVIEWER #2:**

**While the manuscript presents insights into women's domestic workload related to food and family care during the first wave of the COVID-19 pandemic in Chile, there are some significant concerns that need to be addressed.**

**Comment #1. Firstly, the methodology used in the study is too simple and does not provide enough depth to draw meaningful conclusions. The study relies on a Likert scale and an open comment section, which may not be sufficient to capture the complexity of women's experiences during the pandemic adequately.**

**Response:** Thanks to the reviewer for allowing us to reflect on this point. We agree with the reviewer that this Likert scale collects perceptions about different topics, and this could overlook the problem that our study considered. However, it is a suitable methodology for collecting information during the pandemic time, considering the sanitary conditions people were living in (long lockdown period). Additionally, this methodology allowed participants to answer the questionnaire quickly (15 to 20 minutes), which was an advantage. This methodology was a good initial approach to what happened with domestic work and women during times of crisis..

Further studies, including complex methodologies, should be conducted to understand the phenomenon integrally. We have added this reflection to the discussion section.

**Comment #2. The authors developed their own scales but do not in detail describe the process, nor use proper statistical methods of testing and validating the items, and scales. Therefore, the manuscript lacks innovation in terms of the research methods used, which limits its impact.**

**Response:** We thank the reviewers for allowing us to reflect on this point. We have added a paragraph detailing the process of scale creation. Please, refer to the material and methods section.

We recognized the methodology limitations and added them to the discussion section. However, this study gives a first approach to the context regarding domestic work women who lived during a sanitary crisis, which is an innovative situation per se. Further studies could use information from our study as a baseline.

**Comment #3. Also, the study is qualitative, and as it has been three years since the COVID-19 pandemic began, it is unlikely to merit high-impact publication. While qualitative studies can provide valuable insights, they are generally considered less rigorous than quantitative studies and may not have the same impact as more innovative research. At this stage there are hundreds of articles on COVID, why the study is submitted now if the data collection took place in 2020.**

**Response:** Thanks to the reviewer for this comment. Qualitative and Quantitative studies can show different angles on a topic. For our research team, both approaches can give valuable information about a phenomenon.

Even though the pandemic has been almost over in recent months, the results from this study could be a source of information for creating measures to protect women and families from future similar crises. For this reason, the study and its results merit publication. Additionally, we are contributing to the scarce evidence from Latin America.  **Comment #4. You may include some, such as to improve your study:

KABBOUT, R.E. & ZAITER, R. (2022). Covid-19 Related Stressors and Performance: The Case of Lebanese Employees During the Pandemic. International Journal of Management Science and Business Administration, 8(5), 14-25.
Sahni, D.J. (2020). Impact of COVID-19 on Employee Behavior: Stress and Coping Mechanism During WFH (Work From Home) Among Service Industry Employees. International Journal of Operations Management, 1(1), 35-48.
Impact of Social Media, Extended Parallel Process Model (EPPM) on the Intention to Stay at Home during the COVID-19 Pandemic**

**Response:** Thanks to the reviewer for these suggested manuscripts. Due to the relevance to our study topic, we have added two of them to the discussion section (Kabbout & Zaiter, 2022; Sahni, 2020).

**Comment #5. The authors could consider revising the research methods used, incorporating more quantitative data, and adding a more innovative angle to the study to make it more impactful. Alternatively, the authors could consider submitting the manuscript to a journal that is more focused on qualitative research.**

**Response:** Thanks to the reviewer for allowing us to reflect on this topic. We have improved the manuscript according to the comments made by the reviewers. The study is worthy of publication in its current form now. We also think the manuscript fits the journal scope and could be relevant to the readers.
