# Supplementary material for: Food and family care during the COVID-19 pandemic: A study of women’s domestic workload during the first wave in Chile
Source: PLoS One. 2024 May 24;19(5):e0301038. doi: 10.1371/journal.pone.0301038 (PMC11125543; doi:10.1371/journal.pone.0301038)
Supplement: Attachment — Submitted filename: Response to reviewers.docx [file pone.0301038.s004.docx]

**Response to reviewers**

**Journal Requirements**

**Please review your reference list to ensure that it is complete and correct. If you have cited papers that have been retracted, please include the rationale for doing so in the manuscript text, or remove these references and replace them with relevant current references. Any changes to the reference list should be mentioned in the rebuttal letter that accompanies your revised manuscript. If you need to cite a retracted article, indicate the article’s retracted status in the References list and also include a citation and full reference for the retraction notice.**

**Response**: Thanks for this recommendation. We have reviewed our references and we do not have retracted references.

To address the reviewers’ comments, we have added two references (#58 and #61). This change was also mentioned in the cover letter.

**Comments reviewer #4**

**1. This research offers a detailed examination of Chilean women's perceptions and experiences with domestic responsibilities during the initial outbreak of the COVID-19 pandemic, with a particular focus on food preparation and family care tasks. By combining quantitative data from a substantial cohort of 2,047 participants with qualitative feedback gathered through open comments, the study enhances our understanding of the gender-specific effects of the pandemic within household settings. The employment of a mixed-methods strategy, leveraging both a Likert scale survey and thematic analysis of qualitative responses, enables a comprehensive investigation into the subject matter, delivering both wide-ranging and in-depth insights. This investigation draws attention to the increased demands placed on women's workloads and overall well-being during the pandemic, highlighting the critical need for policies and initiatives that are attuned to gender disparities. This is of particular importance to those engaged in policy-making and advocacy aimed at promoting gender equity and improving support structures for women in domestic environments.**

**Response**: Thank you for your thorough review and acknowledgment of our research. We appreciate your recognition of the importance of our study in highlighting the gender-specific impacts of the COVID-19 pandemic on Chilean women's domestic responsibilities. We aimed to provide comprehensive insights by combining quantitative data with qualitative analysis, allowing us to address both wide-ranging and in-depth aspects of the subject matter. Your understanding of the critical need for policies addressing gender disparities is valued, and we hope our research contributes to this goal. Thank you for your feedback.

**2. Although this investigation draws on a broad sample, the method of internet-based recruitment and the self-selection of participants may have biased the sample towards specific demographic groups. In future research directions, authors suggested that efforts be made to include women who may have limited access to the internet and those from a wider array of socioeconomic backgrounds. Additionally, incorporating a comparative analysis between genders in future studies could yield valuable insights. While the study points to the necessity of intervention, offering more explicit guidance on particular policies or initiatives that could alleviate women's burdens would be advantageous. For example, examining the impact of flexible working arrangements, mental health support, and measures to encourage a fair distribution of domestic duties could offer practical guidance for policy makers and community leaders.**

**Response**: Thank you for your suggestion. We have added explicit initiatives to the text. Please refer to lines 452 to 467.
